# Parkinson's Disease Rating Scale Using Synchronization Analysis of Gait Dynamics

**DOI:** 10.1155/2021/5651519

**Published:** 2021-08-25

**Authors:** Naeimehossadat Asmarian, Ahmad Ruzitalab, Gholamhossien Erjaee, Mohammad Hadi Farahi, Seyyed Mojtaba Asmarian

**Affiliations:** ^1^Anesthesiology and Critical Care Research Center, Shiraz University of Medical Sciences, Shiraz, Iran; ^2^Department of Applied Mathematics, Ferdowsi University of Mashhad, Mashhad, Iran; ^3^Think Tank, Iran National Bank, Shiraz, Iran; ^4^Mathematics Department, University of California, Irvine, USA; ^5^Faculty of Engineering, Ardakan University, Ardakan, Iran

## Abstract

Analysis of gait dynamics is a noninvasive and totally painless test, and it can be an ideal method for the diagnosis of neurodegenerative diseases. In this study, based on the strength of synchronization between dynamics of strides, we have suggested a rating scale method for Parkinson's disease (PD). *Methods*. The sample included 15 persons with PD (age: 66.8 ± 10.9 years) and 16 healthy persons (age: 39.3 ± 18.5 years) which were recruited from the Neurology Outpatient Clinic at Massachusetts General Hospital and were instructed to walk a 77 m long, straight hallway. The time interval of strides and subphases of strides were measured. Using the Hilbert transformation method, we obtained the data phase and used mean absolute error (MAE) to calculate the synchronization strength of the data phase. *Results*. In order to check the accuracy of our method, we measured the correlation between our numerical results (MAE) and values of the Hoehn-Yahr scale. Spearman's rank correlation coefficients (*r*) and the *P* values were calculated. MAE of left and right stride intervals (LRSI) significantly correlates with the Hoehn-Yahr scale for the subjects with PD (with *r* = 0.60 and *P* = 0.025 < 0.05). *Conclusion*. We have revealed that the synchronization weakness of LRSI shows the severity of PD. This method seems to be well suited as a rating scale for people with PD.

## 1. Introduction

PD is a degenerative disease of the central nervous system, mainly affecting the motor system. PD motor symptoms result from the death of dopamine-generating cells in the substantia nigra, a region in the midbrain [1]. Early in the course of the disease, the most obvious symptoms are movement related which include shaking, rigidity, slowness of movement, and difficulty in walking and gait [2]. Later on, thinking and behavioral problems may arise, with dementia which commonly occurs in the advanced stages of the disease, and depression is the most common psychiatric symptom [3].

Research studies of PD require a means of rating the severity of the disease by measurement of motor manifestations, assessment of the ability to perform daily functional activities, and symptomatic response to medication. The most common rating scales are the Unified Parkinson Disease Rating Scale (UPDRS), the Hoehn and Yahr (HY) staging, and the Schwab and England rating of activities of daily living. From which, the UPDRS has gained the greatest acceptance as a tool for the evaluation of interventions and as a clinical tool to follow up patients. This rating scale includes four subscales. Subscale 1 covers mentation, behavior, and mood. Subscale 2 rates activities of daily living. Subscale 3 is a clinician rating of the motor manifestations of PD. Subscale 4 covers complications of therapy [4]. The HY staging is probably the most widely known evaluation of people with PD. By this scale, a higher score indicates more advanced disease and includes stages 1 through 5. The modified HY scale is proposed with the addition of stages 1.5 and 2.5 to help describe the intermediate course of the disease [5]. The Schwab and England Scale is an “activities of daily living” (ADL) scale frequently used to provide a single estimate of the patient's ability to function. The rating is done by an examiner interviewing the patient and frequently a collateral source, such as a spouse. This rating varies from 0 to 100% using 5% increments [4]. Although these are the most widely applied rating scales of PD, there are still substantial limitations to these scales that must be considered when using them for research [4]. The clinician should be aware of these different scales and their relative utility. Knowledge of these scales, their validity, their sensitivity to modification, and their specificity and interpretation pitfalls is a prerequisite to good evaluation in daily practice and clinical research.

The authors have considered various analysis tools to diagnose neurodegenerative diseases and to scale their symptoms. Ko and colleagues studied the feasibility of a novel method which is based on breath gas analysis to identify neurodegenerative diseases, especially for PD [6]. Agrawal and Biswas in a short review showed recent advancements in molecular diagnostics particularly biomarkers and imaging spectroscopy for neurological diseases [7]. Sajjadi and his coworkers used diffusion tensor MRI for single-subject diagnosis in neurodegenerative diseases [8]. Oluwafemi and Ibrahim designed an intelligent system to diagnose neurodegenerative diseases [9]. Anderson and MacAskill showed that eye movement laboratory data can provide valuable information about disease severity, progression, or regression in neurodegenerative diseases [10]. Massai and colleagues studied the reliability and validity of the Italian version of the Geriatric Depression Scale in a sample of PD patients [11]. Fereshtehnejad et al. in their study evaluated psychometric properties of the Persian version of the fatigue severity scale to assess fatigue in PD patients [12].

Recently, in health sciences for prognoses and diagnosis, particularly in patients with neurodegenerative conditions, much attention has been placed on the dynamics of gait. Ren and colleagues in their study applied phase synchronization and conditional entropy to the time series pairs of gait rhythms. They revealed that compared with the patients with ALS, HD, and PD, gait rhythms of normal subjects have the strongest phase synchronization property and minimum conditional entropy value [13].

In this article, we show that not only compared with the normal subjects, gait rhythms of subjects with PD have weaker phase synchronization, but also the synchronization strength of gait rhythms of patients with PD is different in illness severity. In fact, we show that for patients with more severe illness, weaker synchronization exists between their left and right strides.

## 2. Materials and Methods

As we will discuss, determining the patient's ability to perform daily tasks, therefore, gait dynamics has an important role in quantifying the severity of the illness. We claim that using synchronization theory in gait dynamics is an effective and efficient method to obtain the severity of PD. Consequently, at first, we introduce the subjects that we have used. Then, knowing that gait dynamics is chaotic, we will use chaos synchronization analysis to measure the severity of the PD.

### 2.1. Subjects

Subjects with PD (*n* = 15 patients, age: 66.8 ± 10.9 (SD) years, 10 men and 5 women) and healthy control (*n* = 16 persons, age: 39.3 ± 18.5 (SD) years, 2 men and 14 women) are the subjects, which were recruited from the Neurology Outpatient Clinic at Massachusetts General Hospital, and the data were provided by Goldberger et al. [14] and downloaded from http://PhysioNet.org. The time series of the stride interval and its two subphases, stance (the time when the foot is on the ground) and swing intervals (the time when the foot is in the air), from the foot switches were recorded. In order to minimize any start-up and end-up effects, we removed the first and last 10 s of each subject's left and right stride interval (LRSI) time series, and a median filter was applied to remove data points that were 3 SD (standard deviation) greater than or less than the median value. These outliers were largely due to the turns at the end of the hallway.

The HY staging has been used to scale the severity of PD. Therefore, to study the accuracy of our scaling method, we have checked the correlation between the output of our method and the results of HY staging.

### 2.2. Method

As it is well known, chaotic systems refer to nonlinear dynamical systems which are very sensitive to initial conditions, in such a way that a small perturbation of these could have unpredictable consequences on the evolution equations. Furthermore, synchronization phenomenon which basically means that two (chaotic) systems oscillate in a same way is an interesting and well-known property of chaotic systems. Indeed, this phenomenon was first discussed by Pecora and Carroll in 1990 [15]. As a kind of this phenomenon, phase synchronization that is observed in systems of various nature [16], including chemical, biological, and physiological systems, has attracted the interest of researchers [17]. In the case of phase synchronization, difference between various states of synchronized systems may not necessarily converge to zero but will stay less than or equal to a constant. Our claim is that there is a correlation between the HY staging of subjects with PD and the strength of synchronization between phases of their LRSI.

#### 2.2.1. Phase of Data

Here, in order to use the synchronization phenomenon, we have used the Hilbert transformation [17] to obtain the phase of data which helps us to distinguish between two different synchronization regimes in the following sense.

An oscillation can be taken as the sine wave *x*(*t*) = *A* sin(*ω*_0_*t* + *ϕ*_0_), where *ω*_0_ denotes the angular frequency which is related to the oscillation period by *ω*_0_ = 2*π*/*T* and should be distinguished from the cyclic frequency of oscillation *f*_0_ = 1/*T*. The intensity of oscillation is determined by its amplitude *A*, and the quantity *ϕ*(*t*) = *ω*_0_*t* + *ϕ*_0_ is called phase. The phase of an oscillator is the key notion and the variable that is of primary importance in the context of synchronization theory. The phase is defined as a quantity that increases by 2*π* within one oscillatory cycle, proportional to the fraction of the period. The phase clearly determines the state of a periodic oscillator; like time, it parameterizes the waveform within the cycle. The phase seems to provide no new information about the system, but its advantage becomes evident if we consider the difference of the phases of two oscillating systems. In this context, suppose that we have an arbitrary narrowband signal, such as respiration signal or measure of stride interval; then, to define its phase *ϕ*(*t*), we may analyze this signal by signal processing which is originally introduced by Gabor. To implement it, one has to construct from the scalar signal *s*(*t*) a complex process:

(1) $$\begin{array}{c} \zeta \left(t\right)=s\left(t\right)+is_{\text{H}}\left(t\right)=A\left(t\right)e^{i\phi \left(t\right)}, \end{array}$$

where the function *s*_H_(*t*) is the Hilbert transform of *s*(*t*) and is defined by

(2) $$\begin{array}{c} s_{\text{H}}\left(t\right)=\pi^{-1}\text{P}.\text{V}.\int_{-\infty }^{+\infty }\frac{s\left(\tau \right)}{t-\tau }d\tau . \end{array}$$

Here, “P.V” means that the integral is taken in the sense of the Cauchy principal value [18]. The instantaneous phase *ϕ*(*t*) and amplitude *A*(*t*) of the signal *s*(*t*) are thus uniquely given via the construction of the analytic signal *ζ*(*t*). Although formally *ϕ*(*t*) and *A*(*t*) can be computed for an arbitrary signal *s*(*t*), they have a physical meaning only if *s*(*t*) is a narrowband signal. Here, we have used this tool to determine the phase *ϕ*(*t*) of the LRSI time series.

#### 2.2.2. Synchronization Strength

After calculating the phase *ϕ*(*t*) of the LRSI time series using the Gabor method, the strength of synchronization between phases of LRSI must be analyzed.

We use a measurement, i.e., mean absolute error (MAE) as a scale for the strength of synchronization of the LRSI phase to emphasize the relation between the strength of synchronization and disease severity. This mean is defined as

(3) $$\begin{array}{c} \text{MAE}=\frac{\sum_{i=1}^{n}\left|{\text{LS}}_{i}-{\text{RS}}_{i}\right|}{n}, \end{array}$$

where LS_*i*_ and RS_*i*_ are the phase of the *i*^th^ left stride and right stride intervals, respectively, and *n* is the number of strides during the experience. We have also considered the synchronization between LRSI and subphases of the strides (e.g., stance and swing) to show that the dynamics of the subphases of the stride is identical to that of the stride itself. To implement this, we have also used

(4) $$\begin{array}{c} {\text{MAE}}_{\text{St}}=\frac{\sum_{i=1}^{n}\left|{\text{LS}}_{i}-{\text{LSt}}_{i}\right|}{n}, \end{array}$$

(5) $$\begin{array}{c} {\text{MAE}}_{\text{Sw}}=\frac{\sum_{i=1}^{n}\left|{\text{LS}}_{i}-{\text{LSw}}_{i}\right|}{n}, \end{array}$$

to obtain the synchronization strength between phases of the *i*^th^ left stride (LS_*i*_) and those of left stance (LSt_*i*_) and left swing (LSw_*i*_), respectively. It can be done similarly for the right stride (RS_*i*_) and right stance (RSt_*i*_) and right swing (RSw_*i*_).

To see and analyze our result, we first determined the phase of our data by using relation (1), and then, to determine the strength of synchronization between LRSI for each subject, we calculated their related MAEs for the phase of data using relations (3)–(5).

## 3. Results

**Figure 1:**
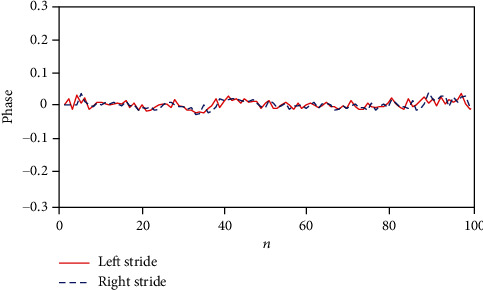
Synchronization between LRSI for the first 100 strides of a healthy control, with MAE = 0.0096.

Figure 1 shows the strong synchronization between the phases of left and right stride time intervals of a healthy control. By calculating the MAE for the phase of healthy control subject data, the result is about MAE = 0.0096. This insignificant error is consistent with Figure 1 (existence of strong synchronization), and this criterion shows the direct relationship between the strength of synchronization and good status of a healthy person.

**Figure 2:**
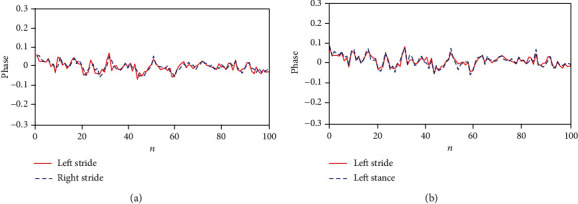
(a) Synchronization of phases of LRSI for the first 100 strides of a low-severity PD person, with HY score = 1.5 and MAE = 0.0131. (b) Between the left stride and left stance, with MAE_St_ = 0.0103.

**Figure 3:**
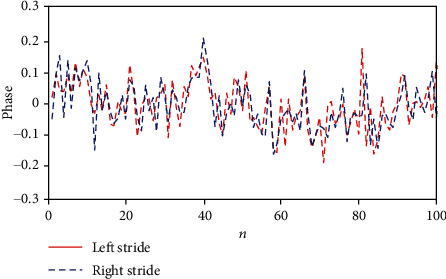
Weak synchronization between phases of LRSI for the first 100 strides of a high-severity PD person, with HY score = 4 and MAE = 0.0583.

Among the subjects with PD, a person who had the best situation with an HY score of 1.5 had the strongest synchronization between his/her LRSI phases and also between phases of his/her left stride interval and left stance, with MAE = 0.0131 and MAE_St_ = 0.0103, respectively (see Figure 2). Conversely, a person with an HY score of 4 had the weakest synchronization between phases of his/her LRSI with MAE = 0.0583 (see Figure 3).

In order to double check the accuracy of the new method, we measured the correlation (statistical relationship) between our numerical results (MAE) and observed data values of the prior scale, Hoehn-Yahr. In order to do so, we calculated Spearman's rank correlation coefficients (*r*) and the *P* values using the SPSS 18.0 software package. All calculated *P* values less than 0.05 were considered statistically significant. More precisely, for the subjects with PD, MAE of LRSI phases significantly correlates with the Hoehn-Yahr scale (with *r* = 0.60 and *P* = 0.025).

## 4. Discussion

There are many rhythms in the human body that play a vital role in human life. Irregular and chaotic rhythms of the heart rate, respiration, gait, and many more have useful information that can determine a person's health or disease status. Some of these rhythms not only are important but also have their harmony with other rhythms. For example, investigating the synchronization of the heart rate and blood pressure with respiration is very important to understand when developing cardiovascular models [18–21]. Synchronization phenomenon which basically means that two (chaotic) systems oscillate in the same way is an interesting and well-known property of chaotic systems. Phase synchronization is a kind of this phenomenon which is observed in chemical, biological, and physiological systems.

Studying dynamics of gait as a chaotic dynamical system in health sciences, particularly in patients with neurodegenerative conditions, can be helpful in the diagnosis and treatment. In human locomotion, not only is the rhythm of each leg important but also the coordination of the two legs. The LRSI time series is affected by the whole nervous system, and their fluctuations may reflect modulation in the underlying mechanism of control. We therefore hypothesized that the strength of synchronization between phases of LRSI for patients with PD would be related to the severity of the disease, and it can result in a new method for PD severity scaling. Hence, in this study, we utilized the strength of phase synchronization of LRSI, obtained by the MAE formula, to determine the severity of the PD. This hypothesis is supported by the findings reported in Results.

Ren et al. applied phase synchronization and conditional entropy to the time series of gait rhythms. Their results showed that compared with the patients with ALS, HD, and PD, gait rhythms of normal subjects have the strongest phase synchronization property and minimum conditional entropy value [13]. However, we have shown in our method that for patients with better status, stronger synchronization exists between their left and right strides; also, each stride synchronizes with its subphases.

This method is simple, cheap, and fast, and especially in the cases where patients' status is severe, this method is more convenient than the prior methods. This can help us to track disease progression and effects of therapeutic interventions. However, this method has its drawbacks. PD symptoms are different for different patients. People are usually more familiar with the movement symptoms of PD. These signs are usually used by doctors to make a diagnosis. But many symptoms of PD are nonmovement and can affect almost any body system and occur any time in the course of the disease (even before movement symptoms) and differ in severity from person to person. The mentioned method is based solely on gait dynamics which is a motor symptom. Of course, by recording more parameters such as handshake level, speech status, and other nonmovement symptoms, a more accurate method can be obtained which is faster, more accurate, and simpler than UPDRS and HY methods and can be a replacement for these methods, and this helps in the detection of PD in subjects with nonmovement symptoms.

Since patients with some other neurodegenerative diseases, like Huntington disease, often display the unsteady walk, the method presented here for scaling severity of PD possibly can be used to scale the severity of such neurodegenerative diseases, and it can be studied in future works.

## 5. Conclusions

Here, we have stated various methods for detecting the severity of PD. We have also presented a method, based on chaos synchronization theory, to show the severity of PD. We have shown that for patients with better status, stronger synchronization exists between their left and right strides and also between strides and subphases of strides. Statistical results confirm the accuracy of our method. Since analysis of gait dynamics is a noninvasive and totally painless test and also this method is cheaper, faster, and more convenient than previous methods, it could be an ideal method for the PD rating scale especially for patients with severe status. Of course, by recording more variables such as handshake level and speech status, a simple and accurate method can be obtained as a replacement for the prior scaling methods.

## Data Availability

The datasets analyzed during the current study are available in https://PhysioNet.org [14]. PhysioBank databases are made available under the ODC Public Domain Dedication and License v1.0.

## Abbreviations

PD: Parkinson's disease
UPDRS: Unified Parkinson's Disease Rating Scale
HY: Hoehn and Yahr
LRSI: Left and right stride intervals
MAE: Mean absolute error.
